# A novel variant BCL11B mutation in a pediatric patient with difficult‐to‐treat eosinophilic esophagitis

**DOI:** 10.1002/jpr3.12139

**Published:** 2024-10-20

**Authors:** Nikita Lalchandani Day, Lauren P. Carlson, Matthew A. Buendia, Girish Hiremath

**Affiliations:** ^1^ Division of Pediatric Gastroenterology, Hepatology, and Nutrition Vanderbilt University Medical Center Nashville Tennessee USA; ^2^ Division of Allergy, Immunology, and Pulmonary Medicine Vanderbilt University Medical Center Nashville Tennessee USA

**Keywords:** BCL11B, difficult‐to‐treat, eosinophilic esophagitis, genetic mutations

## Abstract

Eosinophilic esophagitis (EoE) is an immunoinflammatory disease of the esophagus attributable to a complex interaction between genetic and environmental factors. While several genetic risk variants have been linked with EoE, we report a novel association between B‐cell lymphoma/leukemia 11B genetic mutation in a child with dysmorphic facies, developmental delays, atopic comorbidities, and difficult‐to‐treat EoE. After a prolonged course of EoE and multiple esophagogastroduodenoscopies with biopsies, this patient achieved clinical and histologic remission on a combination of swallowed topical steroids and high‐dose proton pump inhibitor (PPI) therapy. However, her EoE relapsed when we attempted to wean her off PPI, and it was finally controlled after adding PPI back to her regimen. This report underscores the importance of genetic testing in patients with unusual clinical features and difficult‐to‐treat EoE. Relevant to real‐world clinical practice, this case also raises the question of the treatment goals in children with EoE and underlying genetic mutation(s).

## 1 INTRODUCTION

Eosinophilic esophagitis (EoE) is a complex allergen‐mediated disease characterized by esophageal dysfunction and a peak eosinophil count (PEC) of ≥15 eosinophils/high power field (eos/hpf) on esophageal biopsies, and a strong association with other atopic conditions.^1^, ^2^ While our understanding of the pathogenesis of EoE remains incomplete, complex interactions between genetic and environmental factors are implicated in the development of EoE and response to therapy.^3^ Several genetic risk variants have been reported in EoE patients, even though they may not have a challenging clinical course.^4^ Here, we report a novel association between B‐cell lymphoma/leukemia 11B (*BCL11B*) genetic mutation and difficult‐to‐treat pediatric EoE. *BCL11B* is a crucial C2H2 zinc finger transcription factor essential for developing epidermis and T‐cells.^5^ Pathogenic variants in the *BCL11B* gene have been associated with autosomal dominant immunodeficiency‐49, as well as an intellectual developmental disorder with speech delay, dysmorphic facies, and T‐cell abnormalities (IDDSFTA).^6^

## 2 CASE REPORT

**Figure 1:**
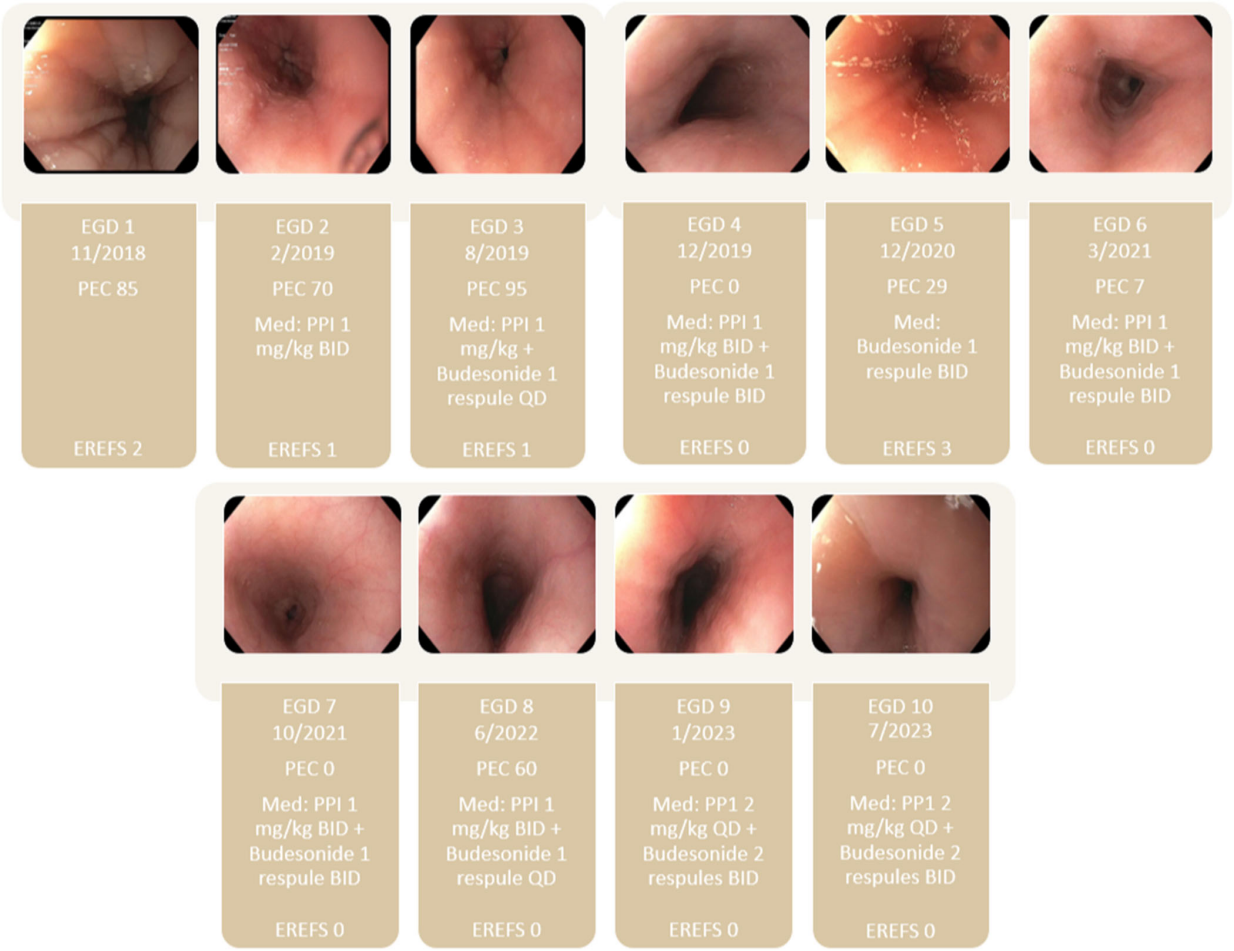
Timeline of endoscopic (EREFS), histologic (PEC), and treatment changes in our patient with *BCL11B* gene mutation and EoE. EGD, esophagogastroduodenoscopy; EREFS, Endoscopic Reference Score; PEC, peak eosinophil count per high power field; PPI, proton‐pump inhibitor.

This patient presented to our clinic when she was 3 years old with chronic feeding intolerance, dysphagia, intermittent vomiting, and fussiness in the setting of dysmorphic facies, developmental delays, severe allergic rhinitis, and mild persistent asthma. Her atopic comorbidities were managed by an Allergist. An esophagogastroduodenoscopy (EGD) was performed to investigate her upper gastrointestinal complaints. The severity of her esophageal mucosal abnormalities assessed per the endoscopic reference score (EREFS) was 2. While her gastric and duodenal biopsies were unremarkable, her esophageal biopsies revealed a PEC of 85 eos/hfp with EoE‐relevant histopathological alterations. A proton pump inhibitor (PPI) therapy (1 mg/kg twice daily [high dose]) was initiated, and this resulted in partial improvement in her symptoms. A follow‐up EGD 3 months later resulted in an improved EREFS score; however, her esophageal biopsies revealed persistent esophageal eosinophilia with a PEC of 70 eos/hpf, suggesting she was not responsive to the high‐dose PPI therapy. At this point, she was diagnosed with EoE per the prevailing guidelines.^7^ Given her limited dietary preferences, her parents and the healthcare team gravitated away from dietary exclusion therapy and opted for pharmacologic options. As such, budesonide (one respule, 0.5 mg/2 mL) slurry was added to her ongoing PPI therapy (combination therapy). At a follow‐up EGD after about 3 months on combination therapy, her esophageal symptoms had resolved; however, her EREFS score remained unchanged, and she continued to have an intense eosinophilic inflammation with a PEC of 90 eos/hpf. As a result, her swallowed budesonide dose was increased to one respule twice daily for 12 months. Eventually, her symptoms completely resolved, and she achieved histologic remission (<15 eos/hpf) on this higher dose of swallowed budesonide and PPI (1 mg/kg twice daily) with greater than 90% compliance to her medications. At this time, her parents requested to minimize her exposure to medications. She was weaned off PPI, but budesonide slurry was continued at the same dose. Due to the coronavirus disease 2019 pandemic and related institutional policies, her follow‐up care was affected for about 12 months. A follow‐up EGD after 12 months had an EREFS score of 3 and revealed a histologic relapse of her EoE with a PEC of 29 eos/hpf. In response, high‐dose PPI (1 mg/kg twice daily) was added back to her EoE therapy plan. Over the next 2 years, she underwent multiple EGDs and failed to achieve sustained histologic remission. Eventually, she achieved complete resolution of her clinical symptoms and attained histologic remission while on budesonide slurry (2 respules twice a day) and PPI (1 mg/kg twice daily) (Figure 1). In parallel, due to her dysmorphic facies and developmental delays, she was evaluated by Genetics, and her whole exome sequencing revealed a pathogenic variant in the *BCL11B* genetic mutation.

## 3 DISCUSSION

Our patient exhibited several key features of IDDSFTA (including global developmental delay, intellectual disability, speech delay, dysmorphic facies, allergies, and asthma). While we report the first case of EoE with *BCL11B* mutation, associations between *BCL11B* gene mutation and other atopic diseases, such as severe asthma and allergies, have been previously reported.^8^, ^9^ Future work should clarify why atopic diseases (including EoE) arise in some individuals with germline *BCL11B* variants but not others, and how specific variants lead to atopy.^10^ Our case underscores the importance of genetic testing in EoE patients who might have unusual clinical features (such as dysmorphic facies and developmental delays) and especially in those who do not respond adequately to commonly used medications. Additionally, this case raises the question of the treatment goals in children with underlying genetic mutation(s) relevant to real‐world clinical practice. The clinical and histological remission guidelines remain variable in research studies and clinical practice. The clinical and histological remission guidelines remain variable in research studies and clinical practice. Additional data are needed to determine if the objective in EoE patients with genetic mutations should be to achieve clinical remission or extend to clinical and histological remission, as is aimed for those without any genetic mutations.

## CONFLICTS OF INTEREST STATEMENT

Girish Hiremath serves as a consultant to Bristol Myers Squibb, Regeneron, and Sanofi. Other authors declare no conflict of interest.
